# A New Era of Neuro-Oncology Research Pioneered by Multi-Omics Analysis and Machine Learning

**DOI:** 10.3390/biom11040565

**Published:** 2021-04-12

**Authors:** Satoshi Takahashi, Masamichi Takahashi, Shota Tanaka, Shunsaku Takayanagi, Hirokazu Takami, Erika Yamazawa, Shohei Nambu, Mototaka Miyake, Kaishi Satomi, Koichi Ichimura, Yoshitaka Narita, Ryuji Hamamoto

**Affiliations:** 1 Division of Medical AI Research and Development, National Cancer Center Research Institute, Tokyo 104-0045, Japan; 2 Cancer Translational Research Team, RIKEN Center for Advanced Intelligence Project, Tokyo 103-0027, Japan; 3 Department of Neurosurgery and Neuro-Oncology, National Cancer Center Hospital, Tokyo 104-0045, Japan; masataka@ncc.go.jp (M.T.); yonarita@ncc.go.jp (Y.N.); 4 Division of Brain Tumor Translational Research, National Cancer Center Research Institute, Tokyo 104-0045, Japan; kichimur@ncc.go.jp; 5 Department of Neurosurgery, Faculty of Medicine, The University of Tokyo, Tokyo 113-8655, Japan; tanakas-tky@umin.ac.jp (S.T.); lmf220316@hotmail.com (S.T.); takami-tky@umin.ac.jp (H.T.); erikakondoyamazawa@gmail.com (E.Y.); shonambu@gmail.com (S.N.); 6 Department of Diagnostic Radiology, National Cancer Center Hospital, Tokyo 104-0045, Japan; mmiyake@ncc.go.jp; 7 Department of Diagnostic Pathology, National Cancer Center Hospital, Tokyo 104-0045, Japan; ksatomi@ncc.go.jp

**Keywords:** multi-omics analysis, machine learning, neuro-oncology, glioma

## Abstract

Although the incidence of central nervous system (CNS) cancers is not high, it significantly reduces a patient’s quality of life and results in high mortality rates. A low incidence also means a low number of cases, which in turn means a low amount of information. To compensate, researchers have tried to increase the amount of information available from a single test using high-throughput technologies. This approach, referred to as single-omics analysis, has only been partially successful as one type of data may not be able to appropriately describe all the characteristics of a tumor. It is presently unclear what type of data can describe a particular clinical situation. One way to solve this problem is to use multi-omics data. When using many types of data, a selected data type or a combination of them may effectively resolve a clinical question. Hence, we conducted a comprehensive survey of papers in the field of neuro-oncology that used multi-omics data for analysis and found that most of the papers utilized machine learning techniques. This fact shows that it is useful to utilize machine learning techniques in multi-omics analysis. In this review, we discuss the current status of multi-omics analysis in the field of neuro-oncology and the importance of using machine learning techniques.

## 1. Introduction

The global incidence rate of brain and nervous system cancers is 4.63 per 100,000 person-years, and they account for 2% of all cancers [1]. Furthermore, it is the most common cause of death in childhood (between 0 and 19 years). Glioblastoma multiforme (GBM), the most malignant primary brain tumor (glioma) according to the World Health Organization (WHO), has the worst prognosis with only 6.8% patients with a 5-year survival rate [2]. Therefore, it is imperative to understand neuro-oncology more deeply and develop effective treatment; paradoxically, this is hindered by the relatively small number of patients [3] and the consecutive little information that can be obtained. One possible solution is making an animal model; however, as there are significant differences in biometrics and anatomy between human and animal models, information derived directly from humans is absolutely essential [4,5,6]. The traditional solution to this problem has been to increase the frequency and resolution of the test. As such, tests can be invasive for the patient, more emphasis has been placed on increasing the resolution of the test. For example, using computed tomography (CT) or magnetic resonance imaging (MRI) instead of radiographs provides a more accurate picture of the tumor. Next-generation sequencing (NGS) is a high-throughput technology that provides more information than immunostaining [7]. The data obtained by these techniques were referred to as “omics data”. Analyzing one type of data is sometimes referred to as single-level omics analysis [8] and has been partially successful. The NGS-based analysis of 324 cancer-associated genes, FoundationOne, is a good example of success [8]. However, single-level omics data analysis has a limitation as it is unclear as to which is the appropriate data type for representing clinical features. Assuming that a tumor is tested for drug resistance, which type of data would predict drug resistance most accurately—DNA sequencing data of the tumor sample, the patient’s blood test data, or MRI? The answer is, as yet, unknown. As drug resistance is the result of a complex biological response, it is difficult to identify data types that may significantly contribute to predict the resistance. For example, as only a subset of GBM patients respond to immunotherapy, researchers are now seeking appropriate biomarkers for the disease [9,10]. One solution to this problem is the use of multi-omics analysis. Even if the appropriate data type required is unknown, the combination of one or more data types may prove useful [11,12]. However, there remains the problem that it is impossible for humans to find principals and make a decision because of too much information. In single-level omics data also, data are in the form of huge matrices that have several hundred rows (of number of patients) and tens of thousands of columns (number of features). In multi-omics analysis, it is possible to integrate some of these matrixes to find laws. This is a difficult problem, but one that can be solved using machine learning techniques. In the field of machine learning, this research is called multi-view learning, where information belonging to different layers can be integrated to find laws [13,14]. Thus, we postulate that the combination of multi-omics analyses is crucial in neuro-oncology research wherein the number of cases is relatively small. It is important to note that with the growing global expectations for the medical application of artificial intelligence, many research results on the application of machine learning and deep learning technologies to medicine have been reported [15,16,17,18,19,20,21,22,23,24], and more than 60 AI-powered medical devices have been approved by the US FDA [25]. Under these circumstances, it is essential to utilize machine learning techniques appropriately in the field of neuro-oncology.

Of note, genomics [26], transcriptomics [27], or high-throughput proteomics [28], are “–omics” approaches providing a substantial dataset of information from comprehensive analysis of molecular substances. Regarding multi-omics analysis, from a broad perspective, multi-omics analysis consists of three parts: input data, methods, and output data. Input data is a starting point and uses for predicting something meaningful. Methods are analysis methods like hierarchical clustering. Output data nearly equal results. To use culinary analogy, input data are fresh ingredients, methods are recipes, and output data are dishes. In this review, we focus on the main outcome and input data types of multi-omics analyses in neuro-oncology, so we discuss ingredients and dishes in detail. Some previous review papers have focused on the method of multi-omics analysis in oncology, but none have focused on output and input data [11,29,30,31,32]. Although focusing on methods is important, output and input are also important for the following reasons. First, it would be possible to count backward from output you seek to the input required, if the right combination of output and input data is known. Furthermore, more importantly, it is essential to extend every concept of omics data. For example, the omics data obtained by molecular biologists are often generated using high-throughput technologies, whereas those by radiologists are often through medical images. No restriction should be placed on the type of data used as input by the researcher as every data type is produced by observing the same condition from a different perspective.

In the first part of this review, we review a brief history of multi-omics analysis in the field of neuro-oncology over the years; in the second, we highlight the current knowledge on multi-omics analysis; and in the third, we discuss the future direction of research.

## 2. A Brief History of Multi-Omics Analysis in Neuro-Oncology

### 2.1. Beginning Of Neuro-Oncology Research and Treatment

The modern accounts of neurosurgery and neuro-oncology begin with Harvey Cushing in the early 1930s [33,34]. In its infancy, surgical resection was the only treatment for brain tumors until a landmark prospective randomized-controlled study on 1,3-bis(2-chloroethyl)-1-nitrosourea (BCNU) and/or radiation therapy was carried out in patients with anaplastic glioma in 1978 [35]. The study’s protocol was very innovative as it was the first trial to determine whether a combination of treatment methods in anaplastic glioma was effective or not. The integration of treatment methods, surgical resection, chemotherapy and radiation therapy were completed for the moment in 2005, by “radiotherapy plus concomitant and adjuvant temozolomide (TMZ) for glioblastoma” [36].

### 2.2. Genotyping

Previously, brain tumors could only be diagnosed by pathological analysis [37]. In 1998, Cairncross et al. discovered that the combined allelic loss of the chromosomes 1p and 19q was associated with both chemosensitivity and longer recurrence-free survival after chemotherapy in patients with anaplastic oligodendrogliomas [38]. This was a monumental time in neuro-oncology research when genetic mutations and treatment responses were linked for the first time. After this, many researchers began to enthusiastically search for clinically meaningful gene alterations. The second breakthrough occurred in 2005. In addition to gene mutation, epigenomic silencing of the O^6^-methylguanine–DNA methyltransferase (*MGMT*) DNA-repair gene was related to good TMZ response in GBM patients [39]. In 2009, the third major discovery in the field occurred, that of isocitrate dehydrogenase 1 gene (*IDH1*) and *IDH2* mutations in glioma patients [40], which showed that a single-point mutation results in the enzymatic activity of the encoded protein and affects tumor malignancy.

### 2.3. Beginning of Multi-Omics Analysis

**Figure 1:**
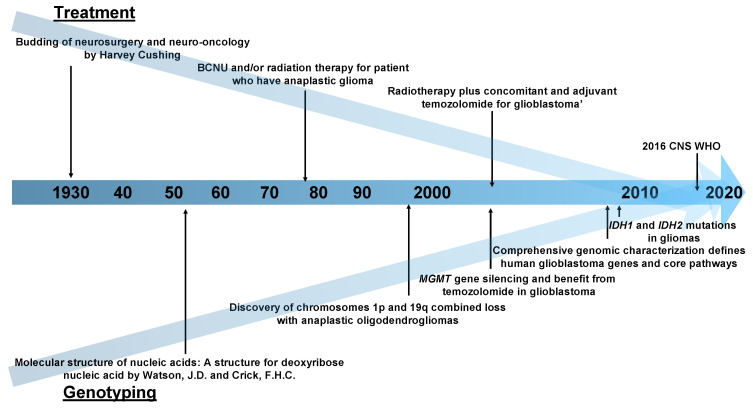
A chronological table of the history of neuro-oncology research. Abbreviations: BCNU, 1,3-bis(2-chloroethyl)-1-nitrosourea; MGMT, O^6^-methylguanine–DNA methyltransferase; IDH, Isocitrate Dehydrogenase; CNS WHO, World Health Organization Classification of the Central Nervous System.

**Figure 2:**
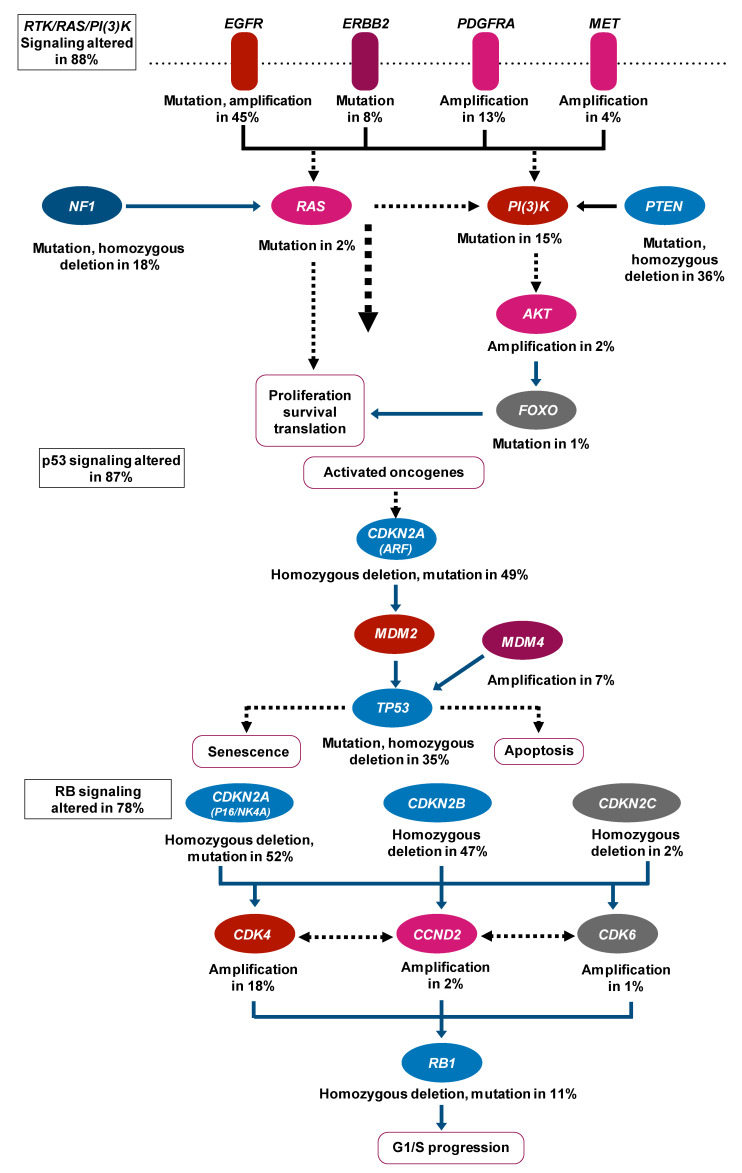
Expected signaling pathway changes in neuro-oncology. This result is achieved by mapping the unequivocal genetic alternations onto major pathways that are already known to be implicated in glioblastoma. Abbreviations: EGFR, Epidermal Growth Factor Receptor; ERBB2, Erb-B2 Receptor Tyrosine Kinase 2; PDGFRA, Platelet-Derived Growth Factor Receptor Alpha; MET, MET Proto-Oncogene, Receptor Tyrosine Kinase; NF1, Neurofibromin 1; PI(3)k, Phosphatidylinositol-3 kinase; PTEN, Phosphatase and Tensin Homolog; AKT, AKT Serine/Threonine Kinas; FOXO, Forkhead Box O; CDKN2A, Cyclin-Dependent Kinase Inhibitor 2A; MDM2, MDM2 Proto-Oncogene; MDM4, MDM4 Regulator of P53; TP53, Tumor Protein P53; CDKN2B, Cyclin-Dependent Kinase Inhibitor 2B; CDKN2C, Cyclin-Dependent Kinase Inhibitor 2C; CDK4, Cyclin-Dependent Kinase 4; CCND2, Cyclin D2; CDK6, Cyclin-Dependent Kinase 6; RB1, RB Transcriptional Corepressor 1.

As mentioned above, the history of treatment in the neuro-oncology field (Figure 1) begins with the validation of the effectiveness of various treatment methods; the integration of these methods is presently underway. The 2016 World Health Organization Classification of the Central Nervous System (2016 CNS WHO) used molecular parameters in addition to histology to define tumor entities probably for the first time [41], i.e., the diagnosis made by the 2016 CNS WHO was an “integrated” diagnosis based on the phenotypic and genotypic classification of the tumor. The paper that can be considered the beginning of this “integration” trend was published in 2008 [42] and reported the interim results of the Cancer Genome Atlas (TCGA) pilot project (Figure 2). It provided a network view of the pathways that were altered in the development of GBM by using DNA copy number and gene expression. This result was achieved by mapping the unequivocal genetic alternations onto major pathways that have been implicated in GBM. Although this paper did not use machine learning methods, there is no doubt that it was a landmark study in multi-omics analysis in neuro-oncology. Also, as described later, it is worth mentioning that most multi-omics studies in neuro-oncology field use the TCGA dataset.

This paper was the starting point for many multi-omics analysis studies in neuro-oncology. The details of these studies are described in the next section.

## 3. Review of Multi-Omics Analysis in the Field of Neuro-Oncology

### 3.1. Search Strategy

We retrieved publications by searching the PubMed database for glioma OR glioblastoma OR medulloblastoma OR meningioma OR schwannoma AND multi omics* OR multiomic* (* means wild-card).

#### 3.1.1. Eligibility Criteria

We selected relevant studies by screening their titles and abstracts, and then reviewed the full texts. We selected papers according to the following criteria.

1. Studies that were not review articles.
2. Studies that were focused on or related to neuro-oncology.
3. Studies that used multi-omics data.

#### 3.1.2. Categories of Papers

We categorized the selected papers according to three main outputs. (i) Pathways and networks—papers that aimed at discovering pathways or networks that were upregulated or downregulated in a tumor situation and included a study point to detect biomarkers; (ii) clinical status—the representative clinical status was prognosis; and (iii) miscellaneous—papers that did not fit into either category were defined as miscellaneous.

### 3.2. Overall Result

Based on the abovementioned criteria, we selected 23 papers (Table 1). The pathways and networks category contained 12 papers, the largest number of papers, the clinical status category contained 7 papers, and 4 papers were categorized as miscellaneous. In terms of the dataset employed, 18 cases, i.e., two-thirds of the total papers, used the TCGA dataset (Supplementary Table S1). The most commonly used input data type was gene expression, which was used in 20 studies. As shown in Supplementary Table S2, the input data styles of copy number change profiles, somatic mutation, and DNA methylation were followed in 13, 12, and 9 studies, respectively. Metabolic profiling, histopathological images, mRNA expression, magnetic resonance imaging (MRI), clinical data, and whole exome sequencing (WES) were used in only one study each. Importantly, most of the papers utilized machine learning techniques to perform regression, classification, clustering, and dimensionality reduction. This fact shows the effectiveness of machine learning techniques in the multimodal analysis of multilayered omics data.

### 3.3. Short Reviews Categorized by Main Outputs

#### 3.3.1. Pathways and Networks

As the paper that was the starting point of multi-omics analysis in neuro-oncology suggested the involvement of new pathways and networks in the disease, it makes sense that the pathway and network analysis category garnered the largest number of papers [42].

Lock et al., using the matrix decompression technique, developed a data decomposing method called joint and individual variation explained (JIVE). Using JIVE, the data were separated into a sum of three terms: a low-rank approximation capturing joint structure between data types, low-rank approximations capturing structure individual to each data type, and residual noise. These predicted the network of gene-miRNA interactions using the loadings of joint components [43].

**Figure 3:**
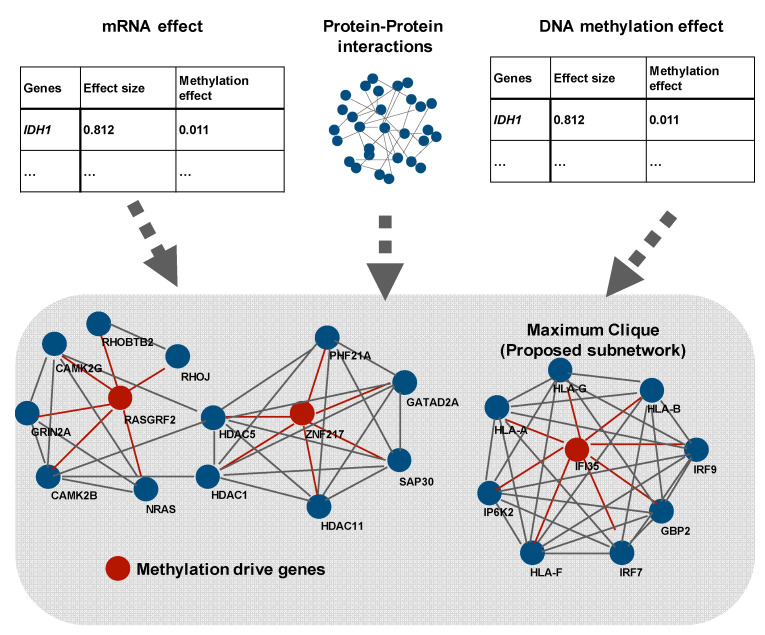
Creation of subnetworks consisting of methylation-driven genes, differentially expressed genes, and known interactions using a network propagation algorithm (modified from Reference [48]). As shown here, some pathway and network studies attempt to discover network genes as nodes and connections between genes as edges. Shafi et al. detected differential expressed genes and methylated genes using the leave-one-out method. Then, they combined the result of the Figure.

Graph theory is often used to discover meaningful pathways and networks [45,48,52] and originated from tools used for analyzing topological problems. In 1736, Swiss mathematician Leonhard Euler introduced the basic idea of graphs, known as the Seven Bridges of Königsberg. Graph theory has been applied in various fields, such as social and information systems, physics, chemistry, and biology as it is useful for representing relationships. A graph consists of nodes (also called vertices or points) and edges (also called links or lines) that connect nodes. When graph theory is applied to biological fields, proteins or genes are often nodes. Zhang et al. proposed a new network analysis method called the prior information-dependent differential network analysis (pDNA), which was based on differential network analysis [48]. The analysis takes into account the following information: (i) a differential edge less likely to exist between two genes that do not participate together in the same pathway; (ii) changes in the networks driven by certain regulator genes that are perturbed across different cellular states; and (iii) the differential networks estimated from multi-view gene expression data that likely share common structures. Zhang et al. applied pDNA by using TCGA (gene expression and copy number change profiles) data to identify the differential networks between the proneural and mesenchymal subtypes of GBM. The results show that four genes were considered as a hub, large degree nodes. *PDGFRA* and *CDK4*, which are often amplified in proneural-type GBM, were included in the four genes. The other example was shown by Shafi et al. (Figure 3). They created a subnetwork that consisted of methylation-driven genes, differentially expressed genes, and known interaction (protein–protein interactions) using a network propagation algorithm [52]. There are several studies that have used original datasets, not TCGA, to provide important results [50,51]. Li et al. identified protein and metabolic markers that correlate to TMZ and discovered a protein-metabolic regulatory network using a mouse GBM model by integrating proteomics and metabolomics. Multi-omics analysis tends to focus on complex mathematical models, but it is important to combine the results of biological experiments and conduct new ones if needed, with mathematical models, as in this study.

#### 3.3.2. Clinical Status

**Figure 4:**
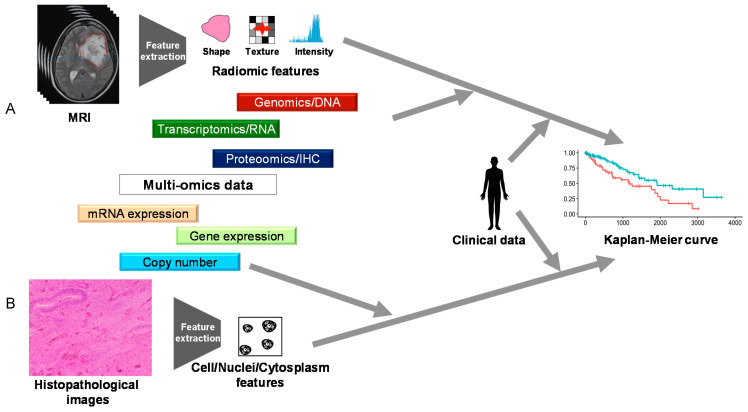
The summary of References [53,54]. Chaddad et al. treated MRI (**A**) and Zhang et al. treated histopathological images (**B**) as data similar to a high-throughput one rather than as merely pictures to obtain clinical data.

Clinical status is often the output of multi-omics analysis. Among clinical status, a prognosis is the most frequent output of multi-omics analysis [44,55,56,57,58,59]. The input data are also mostly derived from TCGA. Two unique studies are presented here (Figure 4). First, Zhang et al. performed an integrated analysis of histopathological images by combining multi-omics data (gene expression, copy number, and mRNA expression data) and clinical data [57]. They handled histopathological images as data, not as a picture, using the open-source software CellProfiler. By doing so, histopathological images could be used as the input data for the machine learning model, similar to that from multi-omics data. Interestingly, they described that combining multi-omics features with histopathological features could predict prognosis more accurately than by using only histopathological features. Chaddad et al. reported a study based on a similar perspective [58]. They used features from MRI, instead of histopathological images, as input data in a machine learning model. They reported that the combination using features from MRI and multi-omics data (genomics, transcriptomics, and proteomics/IHC) marked the maximal area under the curve.

Xiong et al. demonstrated that the average tumor purity that was calculated using multi-omics data by multiple methods correlated with prognosis [59]. This study differs from others in that it indirectly predicts prognosis. Kamoun et al. focused on oligodendroglial tumors [54], not GBM, using an original dataset—the Prise en charge des oligodendrogliomes anaplasiques (POLA) cohort. First, they proved the validity of their integrative clustering techniques, named the cluster of clusters, by showing a strong correlation between the classification result based on their techniques and 1p/19q co-deletion and *IDH* mutation status. Next, they showed three subgroups within 1p/19q co-deleted tumors, that were associated with the specific expression patterns of nervous cell types: oligodendrocyte, oligodendrocyte precursor cell (OPC), and neuronal lineage. Last, they reported that the OPC-like group is associated with more aggressive clinical and molecular patterns, including *MYC* genomic gain, *MAX* genomic loss, *MYC* hypomethylation, and microRNA-34b/c downregulation.

#### 3.3.3. Miscellaneous

The studies categorized in miscellaneous are unique and interesting [61,62,63,64]. A method has been established to create cancer cell lines and animal models from GBM surgical specimens [65,66]. Rosenberg et al. measured and compared the molecular profiles of a set of parental tumors and paired GBM patient-derived cell lines (GBM-PDCLs) by using multi-omics analysis [61]. From their report, overall, the molecular profiles of GBM-PDCLs and paired-parental tumors resemble each other; however, some driver aberrations are lost or gained in the passage from tumor to GBM-PDCLs.

Cancer is a diverse disease, and two people with the same cancer often respond differently to the same stimuli. To answer this question, Bouhaddou et al. tried to build a mechanistic mathematical model that describes the interactions between commonly mutated pan-cancer signaling pathways [62]. They arranged the model to obtain multi-omics data from the MCF10A cell line, a non-transformed mammalian cell line, and trained the model using existing reports and new experimental results to refine biochemical parameters and phenotypic predictions. They reported that their tailored model for glioma could predict an increase in the sensitivity of glioma cell line death to AKT inhibition.

In recent years, neoantigens have received a lot of attention due to their possible role in prognosis and immune therapeutic effect. Nejo et al. evaluated neoantigen expression between primary and recurrent paired 25 glioma samples by using multi-omics data [63].

A study aimed to identify glioma candidate biomarkers using multi-omics analysis was conducted by Liu et al. [64]. The study was characterized by the sheer volume of data and five public datasets. First, these scientists searched for brain-specific biomarkers. Then, they narrowed their search down to those detectable in the cerebrospinal fluid and, finally, they further narrowed their search down to the biomarkers specific to glioma. As a result, they reported that Protein kinase C Gamma (PRKCG) has great potential as a glioma-specific biomarker.

## 4. Discussion and Future Directions

In this review article, we describe the multi-omics analysis in the field of neuro-oncology, focusing on the main output and input data. Although the research on neuro-oncology is increasing, only 23 papers were eligible for our review criteria. The diversity of research is not high and the field is still in its infancy. However, we believe multi-omics analysis will play a central role in precision medicine era for the following reasons. The most important and innovative point of multi-omics analysis is its ability to handle different types of information as parallel and integrate it for human use. In fact, multi-omics analysis is something that human doctors or researchers have been doing unconsciously. Assume that a human doctor predicts the prognosis of a cancer patient. In this case, a skilled doctor would consider not only tumor type and driver gene mutation but also Karnofsky Performance Status, the patient’s medical image, and blood tests, as well as sex, age and familial background. This is because he knows empirically that he can predict more accurately if he takes all of these into account. However, this human-dependent multi-omics analysis has a limitation in that there is no reproducibility and explicability.

Nonetheless, as we have reviewed above, the combination of multi-omics analysis and machine learning could solve this problem. Importantly, judging from the fact that machine learning techniques were utilized in most of the papers presented in this review paper, it can be concluded that machine learning is a useful technique in multi-omics analysis. For this reason, we believe that the following properties of machine learning techniques, which we have previously introduced [67], are important.

1. Multimodal learning: Different types of medical data (genomic, epigenomic data, etc.) can be integrated and treated as input.
2. Multi-task learning: Multiple different tasks can be learned simultaneously by sharing part of the model.
3. Representation learning and semi-supervised learning: Acquiring a representation of the data from a large amount of unlabeled data, which can then be learned from a small amount of labeled data.
4. Automatic acquisition of hierarchical features: Higher-order correlations of inputs can be captured.

What is also expected to become important in the future is research that deals with and integrates information that has not historically been considered analytical data, such as radiological and histopathological images [57,58]. In this review, we have focused on papers on multimodal analysis of omics information such as multilayered genomic information. Recently, radiomics and radiogenomics, which integrate radiological images with clinical information and omics data, have been attracting attention, and successful examples have been published in the field of neuro-oncology [68,69]. There are also some excellent reviews published in the field of radiomics and radiogenomics, which you may be interested in reading [70,71,72].

**Figure 5:**
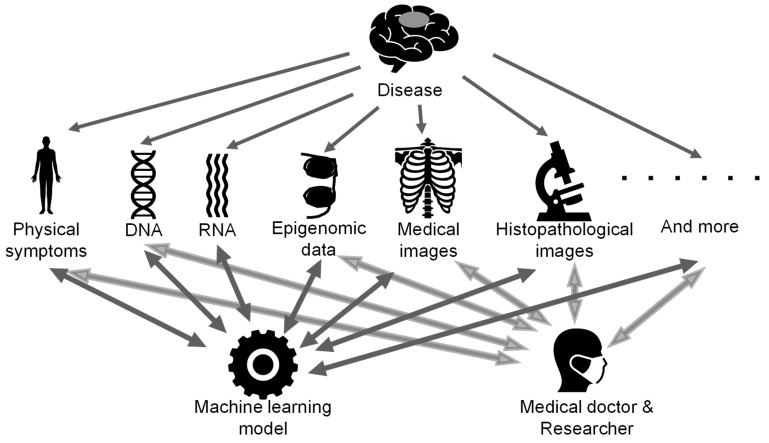
The concept art for future multi-omics analysis. Various types of data are obtained for garnering a better understanding of the nature of the disease and are integrated by machine learning models as well as humans.

One of the most important findings from our survey of various papers is the attempt to reproduce human-dependent multi-omics analysis with machine learning models. All data associated with disease are obtained by observing the disease from different angles. Fragments of the disease have been integrated by humans so far, but they are expected to be integrated by machine learning models in the future (Figure 5). Multi-omics analysis will allow us to understand the nature of the disease more deeply and has the potential to change all medical fields such as drug discovery and therapeutic effects, predict prognosis, and discover the best treatment for each patient [9,11,14,29,30,31,32,67,73,74,75,76,77,78,79,80,81,82]. In addition, as the flow of information is bidirectional, it may be possible to reduce the noise of individual data with integrated information. It has been suggested that integrated data may be more robust than individual data. Thus, multi-omics analysis and machine-learning techniques are just beginning to open the door to a new era in neuro-oncology.

## Figures and Tables

**Table 1:** Summary of the studies short-listed for this review.

No.	Year	Title	Dataset	Input Data Category	Tumor Type	Output Category	Analysis Method
1	2008	Comprehensive genomic characterization defines human glioblastoma genes and core pathways [42]	TCGA	Somatic mutation, copy number change profiles	GBM	Pathway and network	Genomic Identification of Significant Targets in Cancer (GISTIC) algorithm and Genome Topography Scan (GTS) utilizing polynomial regression*
2	2013	Joint and individual variation explained (JIVE) for integrated analysis of multiple data types [43]	TCGA	Gene expression, miRNA expression	GBM	Pathway and network	Joint and Individual Variation Explained (JIVE), which is an extension of PCA* or the SVD* that decomposes the data into low-rank and orthogonal joint and individual components
3	2015	Integrative multi-omics module network inference with Lemon-Tree [44]	TCGA	Gene expression, copy number change profiles	GBM	Pathway and network	The module network method, a special type of Bayesian network* algorithms, with Lemon-Tree
4	2015	Identifying core gene modules in glioblastoma based on multilayer factor-mediated dysfunctional regulatory networks through integrating multi-dimensional genomic data [45]	TCGA	Gene expression, copy number change profiles, somatic mutation, DNA methylation, miRNA expression	GBM	Pathway and network	Core Modules Driving Dysregulation in cancer (CMDD) using PLSR*
5	2016	Causal mechanistic regulatory network for glioblastoma deciphered using systems genetics network analysis [46]	TCGA	Somatic mutation, gene expression, miRNA expression	GBM	Pathway and network	Systems Genetics Network Analysis (SYGNAL) pipeline using cMonkey2 biclustering algorithm*
6	2016	MONGKIE: an integrated tool for network analysis and visualization for multi-omics data [47]	TCGA	Somatic mutation, copy number change profiles	GBM	Pathway and network	Modular Network Generation and Visualization with Knowledge Integration Environments (MONGKIE) using graph clustering*
7	2017	Incorporating prior information into differential network analysis using non-paranormal graphical models [48]	TCGA	Gene expression, copy number change profiles	GBM	Pathway and network	Prior information-dependent differential network analysis (pDNA) using GGM*
8	2017	A systemic analysis of transcriptomic and epigenomic data to reveal regulation patterns for complex disease [49]	TCGA	Gene expression, DNA methylation, miRNA expression	GBM	Pathway and network	Integrative analysis framework by incorporating sparse model, multivariate analysis, elastic net penalized regression*, GGM*, and network analysis
9	2018	Repression of Septin9 and Septin2 suppresses tumor growth of human glioblastoma cells [50]	GEO + cell line	Gene expression, protein expression	GBM	Pathway and network	Multiple analyses combining GBM expression studies from the GEO repository
10	2019	Integrated proteomic and metabolomic profiling the global response of rat glioma model by temozolomide treatment [51]	Mouse model (cell line)	Protein expression, metabolomic profiling	GBM	Pathway and network	Ingenuity pathway analysis
11	2019	A multi-cohort and multi-omics meta-analysis framework to identify network-based gene signatures [52]	TCGA, GEO, CGGA	Gene expression, DNA methylation	GBM, LGG	Pathway and network	Multi-cohort and multi-omics meta-analysis framework using perturbation clustering*
12	2020	Identifying cancer driver lncRNAs bridged by functional effectors through integrating multi-omics data in human cancers [53]	TCGA	Gene expression, copy number change profiles, somatic mutation, DNA methylation, miRNA expression	GBM	Pathway and network	DriverLncNet is proposed to integrate multi-omics data to identify lncRNAs as drivers of human cancer using PLSR*
13	2016	Integrated multi-omics analysis of oligodendroglial tumors identifies three subgroups of 1p/19q co-deleted gliomas [54]	POLA	Gene expression, DNA methylation, miRNA expression	OT	Clinical status	k-means clustering*
14	2018	Whole-genome multi-omic study of survival in patients with glioblastoma [55]	TCGA	Gene expression, DNA methylation, somatic mutation, copy number change profiles	GBM	Clinical status	Multi layered Bayesian regression*
15	2019	Group lasso regularized deep learning for cancer prognosis from multi-omics and clinical features [56]	TCGA	Gene expression, copy number change profiles, somatic mutation, protein expression	GBM	Clinical status	Group lasso regularized deep learning*
16	2019	A novel MKL Method for GBM prognosis prediction by integrating histopathological image and multi-omics data [57]	TCGA	Histopathological images, gene expression, copy number change profiles, mRNA expression	GBM	Clinical status	Multiple kernel learning*
17	2020	Integration of radiomic and multi-omic analyses predicts survival of newly diagnosed *IDH1* wild-type glioblastoma [58]	TCIA, TCGA, MUHC	MRI, gene expression, somatic mutation, clinical, protein expression	*IDH1* wild-type GBM	Clinical status	Random forest*
18	2020	Multi-dimensional omics characterization in glioblastoma identifies the purity-associated pattern and prognostic gene signatures [59]	TCGA, GEO, CGGA	Gene expression, copy number change profiles, somatic mutation, DNA methylation	GBM	Clinical status	LASSO*
19	2020	Integrating genomic data with transcriptomic data for improved survival prediction for adult diffuse glioma [60]	TCGA	Gene expression, DNA methylation, somatic mutation, copy number change profiles	GBM, LGG	Clinical status	Random forest*
20	2017	Multi-omics analysis of primary glioblastoma cell lines shows recapitulation of pivotal molecular features of parental tumors [61]	Private dataset	Gene expression, somatic mutation, copy number change profiles	GBM	Miscellaneous	Global Parameters Hidden Markov Model (GPHMM) algorithm*
21	2018	A mechanistic pan-cancer pathway model informed by multi-omics data interprets stochastic cell fate responses to drugs and mitogens [62]	Cell line	Gene expression, copy number change profiles, protein expression	GBM	Miscellaneous	LASSO* and support vector machine*
22	2019	Reduced neoantigen expression revealed by longitudinal multiomics as a possible immune evasion mechanism in glioma [63]	Private dataset	Gene expression, WES	GBM, LGG	Miscellaneous	NetMHCpan using artificial neural networks*
23	2020	Computational identification and characterization of glioma candidate biomarkers through multi-omics integrative profiling [64]	GTEx, TCGA, CGGA, GEO, Ivy GAP	Gene expression, DNA methylation, somatic mutation, protein expression	Glioma	Miscellaneous	Computational integrative multi-omics data analysis

Abbreviations: ML, Machine Learning; TCGA, The Cancer Genome Atlas; TCIA, The Cancer Imaging Archive; GEO, Gene Expression Omnibus; GTEx, Genotype-Tissue Expression; CGGA, Chinese Glioma Genome Atlas; LGG, Lower-Grade Glioma; GBM, Glioblastoma multiforme; POLA, Prise en charge des OLigodendrogliomes Anaplasiques; OT, Oligodendrogial Tumors; MUHC, McGill University Health Centre; Ivy GAP, Ivy Glioblastoma Atlas Project; PCA, Principle Components Analysis; SVD, Singular Value Decomposition; PLSR, Partial Least Squares Regression; GGM, Gaussian Graphical Model. *: Machine learning method.

## Supplementary Materials

The following are available online at https://www.mdpi.com/article/10.3390/biom11040565/s1, Table S1: Studies reviewed in Table 1 grouped by input data category. Table S2: Studies reviewed in Table 1 grouped by input dataset.
