# Supplementary material for: A New Era of Neuro-Oncology Research Pioneered by Multi-Omics Analysis and Machine Learning
Source: Biomolecules. 2021 Apr 12;11(4):565. doi: 10.3390/biom11040565 (PMC8070530; doi:10.3390/biom11040565)
Supplement: Supplementary file 1 [file biomolecules-11-00565-s001.pdf]

Supplementary Materials

Table S1. Studies reviewed in Table 1 grouped by input data category

Input data category	Total Number
Somatic mutation	12
Copy number change profiles	13
Gene expression	20
miRNA expression	6
DNA methylation	9
Protein expression	6
Metabolomic profiling	1
Histopathological images	1
mRNA expression	1
MRI	1
Clinical	1
WES	1

Abbreviations: MRI, magnetic resonance imaging; WES, whole exome sequencing.

Table S2. Studies reviewed in Table 1 grouped by input dataset

Dataset	Total number
TCGA	17
GEO	4
cell line	3
CGGA	2
POLA	1
TCIA	1
MUHC	1
CGGA	1
Private dataset	2
GTEx	1
Lvy GAP	1

Abbreviations: TCGA, The Cancer Genome Atlas; TCIA, The Cancer Imaging Archive; GEO, Gene Expression Omnibus; GTEx, Genotype-Tissue Expression; CGGA, Chinese Glioma Genome Atlas; POLA, Prise en charge des OLigodendrogliomes Anaplasiques; MUHC, McGill University Health Centre; Ivy GAP, Ivy Glioblastoma Atlas Project.
